# Toward Standardized Massive Transfusion Protocols: A Multicenter Evaluation of Practice Variability Within a National Trauma System

**DOI:** 10.3390/healthcare13151848

**Published:** 2025-07-29

**Authors:** Dongmin Seo, Junsik Kwon, Inhae Heo, Younghwan Kim, Jae Hun Kim, Taegyun Kim, Hangjoo Cho, Kyoungwon Jung

**Affiliations:** 1 Division of Trauma Surgery, Department of Surgery, Ajou University School of Medicine, Suwon 16499, Republic of Korea; suh.d.min@gmail.com (D.S.); aquaestel@gmail.com (J.K.); nareun104@hotmail.com (I.H.); 2 Department of Surgery, National Medical Center, Seoul 04564, Republic of Korea; galgunbam2@hanmail.net; 3 Department of Trauma Surgery and Surgical Critical Care, Pusan National University Hospital, Pusan National University School of Medicine, Busan 43241, Republic of Korea; drtrauma73@gmail.com; 4 Department of Emergency Medicine, Seoul National University College of Medicine, Seoul 04564, Republic of Korea; kimtaegyun@snuh.org; 5 Department of Trauma Surgery, Uijeongbu St. Mary’s Hospital, College of Medicine, The Catholic University of Korea, Seoul 04564, Republic of Korea; surgeryman@catholic.ac.kr

**Keywords:** massive transfusion protocol, trauma hemorrhage, trauma centers, blood product administration, performance improvement

## Abstract

**Background/Objectives**: Hemorrhage remains a leading cause of early mortality in trauma patients, and timely transfusion guided by a structured massive transfusion protocol (MTP) is critical for improving outcomes. Although regional trauma centers have been established, standardized MTPs remain insufficiently developed in many settings. This study aimed to evaluate current MTP practices across five major trauma centers within a national trauma care system. **Methods**: Participating institutions provided written protocols and completed a structured survey addressing key domains, including activation criteria, transfusion strategies, laboratory monitoring, adjunct therapies, termination processes, and performance improvement measures. Findings were analyzed and compared against established international recommendations. **Results**: All centers had implemented MTPs and were capable of delivering initial blood products within 15 min. However, considerable variation was observed in activation triggers, transfusion ratios, and laboratory monitoring protocols. None of these centers maintained thawed plasma or whole blood in immediate readiness. Only one of five centers had a formal performance improvement monitoring system. Tranexamic acid was included in all institutional protocols. **Conclusions:** This review highlights significant variability and critical gaps in MTP implementation across trauma centers. Inconsistent activation criteria, the absence of essential components, and limited quality monitoring may compromise the efficacy of current practices. To improve patient outcomes, a standardized, evidence-based MTP framework should be developed and implemented nationwide.

## 1. Introduction

Hemorrhage is a leading cause of death in trauma patients, accounting for approximately 30–40% of trauma-related mortality. Bleeding-related deaths represent the largest proportion of early trauma fatalities [1]. In hemodynamically unstable patients, rapid hemorrhage control is essential. As part of damage control resuscitation, prompt administration of blood products with a balanced transfusion of red blood cells (RBCs), plasma, and platelets is recommended [2], as this approach mitigates traumatic coagulopathy and reduces mortality.

The traditional definition of massive transfusion, the administration of ≥10 units of RBCs within 24 h, has limitations, particularly because it requires survival for at least 24 h and may introduce bias by excluding patients who succumb earlier. Recent systematic reviews have proposed alternative definitions, such as the transfusion of 3–5 units of RBCs within 1–6 h [3]. The Joint United Kingdom Blood Transfusion and Tissue Transplantation Services define massive transfusion based on estimated blood loss; however, accurately quantifying blood loss in clinical settings remains challenging.

To address these limitations, most trauma centers employ massive transfusion protocols (MTPs) [4]. These protocols are executed by multidisciplinary teams that include blood bank personnel, pathologists, trauma surgeons, anesthesiologists, nurses, support staff, and quality improvement specialists. MTP activation prompts the blood bank to prepare and dispatch pre-specified blood products while cross-matching is underway [5]. This process facilitates rapid transfusion during initial resuscitation, enhances efficiency, reduces the total transfusion volume, and lowers associated costs [6].

Evidence supports the use of balanced transfusion strategies to improve outcomes in patients requiring massive transfusion. The Prospective, Observational, Multicenter, Major Trauma Transfusion study demonstrated that higher ratios of plasma and platelet transfusion were associated with improved 24 h survival [7]. The subsequent Pragmatic, Randomized Optimal Platelet and Plasma Ratios (PROPPR) study showed that a 1:1:1 transfusion ratio of RBCs, plasma, and platelets reduced 24 h mortality due to exsanguination [8]. As a result, this ratio has become the standard in MTPs. These protocols aim to promptly identify patients who require a massive transfusion, ensure balanced blood product delivery, and promote efficient and safe transfusion practices [9]. Ongoing efforts continue to refine MTPs in light of emerging evidence and evolving clinical practices [5,10].

In South Korea, the government established 17 regional trauma centers between 2012 and 2022 [11]. Although these centers are not formally tiered as in the United States, they serve functions comparable to Level 1 trauma centers and play a central role in regional trauma care. Most have implemented MTPs developed by benchmarking established international models and adapting them to the Korean healthcare context.

The present study aimed to analyze the MTPs employed by five major trauma centers in South Korea and propose a foundational framework for standardized MTP implementation that incorporates current best practices.

## 2. Materials and Methods

MTPs are standardized institutional procedures designed to ensure the timely delivery of blood products to patients with life-threatening hemorrhage. These protocols typically include predefined criteria for activation, a fixed-ratio transfusion strategy involving red blood cells, fresh-frozen plasma, and platelets, as well as guidance on laboratory monitoring, adjunctive therapies, and termination of the protocol [12].

This study was conducted as part of the prospective multicenter Cohort for Optimal Hemostatic Transfusion in Trauma–Korea (COHTRA-K) project, which aims to establish optimal transfusion strategies for patients with massive traumatic hemorrhage. Four regional trauma centers and one high-level trauma treatment center in the Republic of Korea participated in this initiative. MTPs actively used in clinical practice at each institution were collected for evaluation. The analytical framework was based on the evaluation tool presented in the ACS TQIP Massive Transfusion in Trauma Guidelines, developed by the American College of Surgeons (ACS) Committee on Trauma under the Trauma Quality Improvement Program (TQIP).

Designated personnel who oversee MTP implementation at each center completed a structured questionnaire developed by the COHTRA-K research group. The questionnaire addressed seven core domains: essential components, initiation, fixed-ratio blood component batch, laboratory monitoring, adjuncts, termination, and performance improvement monitoring. The content of the questionnaire was finalized through multiple research team meetings to ensure comprehensive coverage of documentation practices, operational procedures, and institutional resources. The full questionnaire is provided in the Supplementary Material.

Responses were compiled and systematically analyzed to compare the structure and application of MTPs across the five participating centers. Each response item was cross-referenced to assess how MTPs were implemented in cases of massive hemorrhage, enabling an objective representation of current practices.

## 3. Results

Table 1, Table 2, Table 3, Table 4, Table 5, Table 6 and Table 7 summarize the presence or absence of each MTP component across five centers. In these tables, ‘Y’ indicates that the item is implemented at the institution, while ‘N’ denotes that it is not.

### 3.1. Essential Components

All five trauma centers (A–E) incorporated the core elements of an MTP, including activation criteria, balanced transfusion strategies, operating room transition, therapeutic targets, adjunctive treatments, and defined termination procedures. Four centers provided explicit protocols for transferring patients to an Angio Suite or intensive care unit, whereas one center lacked such guidance. Only one center had implemented a structured performance improvement (PI) monitoring system; the remaining centers reported no formal PI measures (Table 1).

### 3.2. Initiation of MTP

Each center maintained written criteria for MTP activation. Common triggers included systolic blood pressure below a designated threshold, elevated heart rate, a positive Focused Assessment with Sonography for Trauma, or penetrating trauma. Four centers employed a scoring system (e.g., the Assessment of Blood Consumption [ABC] score) above a defined cutoff, while one center relied solely on hypotension. Several centers noted that persistent hemodynamic instability could prompt activation based on clinical judgment. All centers reported mechanisms to ensure delivery of the first blood product within 15 min of MTP initiation (Table 2).

### 3.3. Fixed-Ratio Blood Component Batches and Unmatched Blood

All five centers initially administered type O RBCs, with two also using AB plasma in early resuscitation. No center utilized thawed plasma or low-titer group A plasma. Platelet units were not included in initial transfusion packs at any site. Three centers maintained on-site blood product storage within the trauma bay or emergency department (ED), whereas two lacked immediate access. Four centers employed blood warmers or rapid infusion devices; one center did not report using any specialized equipment. All centers followed fixed RBC-to-plasma ratios in early transfusion (Table 3).

### 3.4. Laboratory Monitoring

Four centers outlined defined testing intervals and laboratory parameters for MTP management; one did not specify a schedule. All centers routinely assessed the prothrombin time/international normalized ratio, activated partial thromboplastin time, fibrinogen levels, and platelet counts. However, one center did not monitor hemoglobin, hematocrit, or lactate levels. Viscoelastic testing using thromboelastography or rotational thromboelastometry was available in three centers, whereas two lacked this capacity (Table 4).

### 3.5. Adjuncts

Tranexamic acid (TXA) was included in the MTPs of all five centers. Cryoprecipitate was available for patients with severe coagulopathy. One center used fibrin concentrate or prothrombin complex concentrate, whereas four did not report the use of these agents (Table 5).

### 3.6. Termination of MTP

All centers documented criteria and timing for MTP termination and indicated a transition to goal-directed therapy upon protocol completion. However, none provided specific numeric thresholds for discontinuation (Table 6).

### 3.7. PI Monitoring

Only one center reported maintaining a structured PI system, which monitored transfusion times, departmental notification rates at protocol termination, and blood product wastage. The remaining centers had no formalized performance monitoring practices (Table 7).

## 4. Discussion

### 4.1. Summary of Key Findings and Comparison with the Literature

This review of MTPs across five major trauma centers in South Korea revealed substantial variability in protocol components and identified key gaps relative to established best practices. All participating centers had implemented MTPs incorporating core elements such as initiation criteria, balanced transfusion strategies, and termination guidelines. However, differences were observed in activation criteria, transfusion ratios, and blood product logistics. Notably, none of the centers maintained pre-thawed plasma or low-titer O whole blood (LTOWB) for immediate use. Despite these limitations, all centers aimed to deliver blood products within 15 min of MTP activation. Only one center reported a formal performance improvement system to evaluate MTP effectiveness. These findings indicate that while MTPs are in routine use, their standardization and optimization remain incomplete.

Previous studies have highlighted wide variability in the structure and implementation of MHPs among Level 1 trauma centers in both North America and Europe [13,14,15]. These reports emphasize discrepancies in activation criteria, transfusion practices, adjunct use, and performance monitoring across institutions.

### 4.2. Differences and Similarities with ACS TQIP Guidelines

Comparison with the ACS TQIP guidelines revealed several discrepancies. The ACS TQIP outlines a comprehensive MTP framework, including clear activation criteria, immediate availability of blood products, balanced transfusion ratios, adjunctive therapies, termination criteria, and mechanisms for performance monitoring [12,16]. For activation, objective criteria, such as an ABC score ≥ 2 or clinical evidence of hemorrhagic shock, are recommended [12]. Early activation is emphasized, as it has been associated with improved survival outcomes [7,17].

Blood product availability and transfusion ratios also diverged from ACS TQIP recommendations. The guidelines advocate a 1:1:1 ratio of packed red blood cells (PRBCs), fresh frozen plasma (FFP), and platelets [18]. However, none of the surveyed Korean centers maintained thawed plasma on-site, resulting in an unbalanced transfusion approach during initial resuscitation. This limitation is largely attributable to the absence of national usage guidelines and clinical consensus on thawed plasma, as well as insufficient infrastructure, including storage and management systems. This delay contradicts ACS TQIP guidance, which recommends immediate availability of thawed plasma [19]. Institutions maintaining ED plasma inventories have reported reductions in product wastage and coagulopathy [20].

A further distinction is the absence of whole blood use. Evidence suggests that LTOWB improves survival in patients with severe hemorrhage when compared to component therapy [21]. Despite this, no Korean trauma center had incorporated LTOWB into their MTP. In Korea, this limitation is primarily attributable to the lack of nationally established protocols and clinical consensus for LTOWB use, along with the absence of a structured system for its collection and distribution. Adjunctive therapies recommended by ACS TQIP were variably implemented. All centers included TXA in their protocols, and three utilized viscoelastic hemostatic assays to guide resuscitation [8,22].

Despite variations in specific practices, the MTPs used in Korean trauma centers share several structural similarities with the ACS TQIP recommendations. All centers have implemented protocols encompassing key elements such as activation, transfusion strategy, and termination. A fixed-ratio transfusion approach, reflecting the concept of balanced resuscitation, was consistently applied across institutions. Additionally, all centers maintained written criteria for MTP activation, aligning with the TQIP emphasis on standardized and objective activation protocols.

### 4.3. MTP Activation, Blood Product Use, and Adjuncts

Our findings are consistent with prior Korean studies. A single-center report noted that following MTP implementation, the median time to first transfusion decreased from approximately 41 min to 15 min [19]. A multicenter analysis demonstrated that each minute of delay in delivering the first blood product after MTP activation increases the odds of mortality by 5% [23].

Balanced transfusion has been consistently associated with improved outcomes. Both retrospective analyses and randomized trials, including the PROPPR study, support early administration of high ratios of FFP and platelets relative to PRBCs [8,18]. Korean studies similarly reported that higher FFP and platelet ratios were associated with reduced mortality [24,25]. Internationally, the majority of trauma centers participating in the ACS TQIP have adopted MTPs aligned with damage control resuscitation principles [26].

The universal use of TXA across all surveyed centers aligns with evidence supporting its survival benefit when administered early in hemorrhagic trauma. In contrast, the absence of LTOWB use in Korea stands in opposition to evolving practices in U.S. and European trauma systems, where whole blood programs have been associated with improved outcomes [21].

Although mixed results have been reported regarding the mortality benefit of MTP implementation, most studies demonstrate improvements in morbidity and process-related measures [20,27]. Centers that adhere more closely to evidence-based MTP guidelines have shown better outcomes, including reduced coagulopathy and improved survival [19].

### 4.4. PI Monitoring

A key area for improvement is the implementation of formal performance monitoring. The ACS TQIP specifically recommends that MTPs include quality assurance components [12]. Relevant performance indicators include appropriate MTP activation, time to first transfusion, achieved blood product ratios, incidence of coagulopathy, and overall patient outcomes [23,28]. In this study, only one center reported an active performance monitoring system.

We recommend that all Korean trauma centers incorporate massive transfusion case reviews into their quality assurance programs. Key metrics should include appropriate MTP activation, time to delivery of blood products, PRBC–FFP–platelet ratios at 4 and 24 h, incidence of acute traumatic coagulopathy, blood product wastage, transfusion ratios at the point of hemorrhage control, and documentation of regular multidisciplinary case reviews. To implement PI with minimal burden, we propose designating a charge person responsible for MTP oversight at each center and developing generalized, tailored templates utilizing these key metrics.

### 4.5. Limitations

This study had some limitations. First, the analysis was based on written protocols and survey responses rather than direct observation of clinical practice, which may not fully reflect real-world implementation. Prospective data collection on MTP protocols and associated clinical outcomes will facilitate future investigations into their relationships with mortality and transfusion requirements. Second, the heterogeneity in protocol formatting across institutions introduced challenges in comparative analysis. The sample size was limited to five major trauma centers, which, although representative of high-level trauma care in South Korea, may not capture practices in smaller or less-resourced facilities. Third, the evaluation framework relied primarily on ACS TQIP guidelines, which may not be entirely applicable in the Korean healthcare context due to differing resource availability. Finally, clinical outcome data were not collected, precluding an assessment of protocol effectiveness on patient morbidity or mortality.

### 4.6. Conclusions and Future Directions

Despite these limitations, this multicenter review demonstrates that MTPs have been implemented in all five surveyed regional trauma centers in South Korea, representing a substantial advancement in the national trauma care infrastructure. However, the findings also underscore persistent inconsistencies and deviations from internationally recognized best practices. Notable deficiencies include the absence of readily available thawed plasma, lack of LTOWB, variability in activation criteria, and incomplete integration of adjunctive therapies and monitoring tools. Critically, only one center had established a performance improvement monitoring system. These gaps may compromise the effectiveness of current MTPs and hinder optimal patient outcomes. The Korean experience highlights the challenges of protocol standardization in rapidly evolving trauma systems and provides insights relevant to other countries undergoing similar transitions.

## Figures and Tables

**Table 1:** Presence of essential components in massive transfusion protocols across five trauma centers.

Component	A	B	C	D	E
Initiation	Y	Y	Y	Y	Y
Balanced transfusion	Y	Y	Y	Y	Y
Transition to OR	Y	Y	Y	Y	Y
Transition to Angio Suite	Y	Y	N	Y	Y
Transition to ICU	Y	Y	N	Y	Y
Target	Y	Y	Y	Y	Y
Adjuncts	Y	Y	Y	Y	Y
Termination	Y	Y	Y	Y	Y
PI monitoring	Y	N	N	N	N

OR, operating room; ICU, intensive care unit; PI, performance improvement.

**Table 2:** Criteria and processes for initiation of massive transfusion protocols.

Parameter	A	B	C	D	E
Is the activation criterion documented?	Y	Y	Y	Y	Y
Are objective criteria used for activation? (open-ended)	SBP ≤ 90	SBP ≤ 90	SBP ≤ 90	SBP ≤ 90	SBP ≤ 90
	HR ≥ 120		HR ≥ 120	HR ≥ 120	HR ≥ 120
	Positive FAST		Positive FAST	Positive FAST	Positive FAST
	Penetrating torso injury		Penetrating torso injury	Penetrating torso injury	Penetrating torso injury
Is a large-volume hemorrhage/transfusion predictive score used? (open-ended question)	ABC score ≥ 2	N	ABC score ≥ 2	ABC score ≥ 2	ABC score ≥ 2
Is subjective clinical judgment used?	Persistent hemodynamic instability	Y	N	Y	Y
Is active bleeding requiring surgery or angioembolization included as a criterion?	Y	Y	Y	N	Y
Is transfusion in the trauma bay used as an activation criterion?	N	N	N	N	N
Is there a separate, rapid decision-making process for immediate MTP activation?	Y	Y	Y	Y	Y
Are initial blood products available within 15 min of activation?	Y	Y	Y	Y	Y

SBP, systolic blood pressure; HR, heart rate; FAST, Focused Assessment with Sonography for Trauma; ABC, Assessment of Blood Consumption; MTP, massive transfusion protocol.

**Table 3:** Fixed-ratio blood component practices in initial transfusion batches across five trauma centers.

Component	A	B	C	D	E
Blood preparation in trauma bay/ER	Y	Y	N	N	Y
Use of O (Rh+) RBC	Y	Y	Y	Y	Y
Use of O (Rh−) RBC	N	Y	Y	N	N
Use of AB (Rh+) FFP	Y	N	Y	N	N
Use of low-titer A FFP	N	N	N	N	N
Use of thawed plasma	N	N	N	N	N
Inclusion of platelets in the first transfusion batch	N	N	N	N	N
Storage of platelets in the trauma bay	N	N	N	N	N
Use of a blood warmer	Y	N	Y	Y	Y
Use of a rapid infusion device	Y	N	Y	Y	Y
Enforced fixed RBC-to-plasma transfusion ratio	Y	Y	Y	Y	Y

ER, emergency room; RBC, red blood cell; FFP, fresh frozen plasma.

**Table 4:** Laboratory monitoring parameters specified in massive transfusion protocols.

Parameter	A	B	C	D	E
Type and frequency of laboratory monitoring specified	Y	N	Y	Y	Y
PT (INR)	Y	Y	Y	Y	Y
aPTT	Y	Y	Y	Y	Y
Fibrinogen	Y	Y	Y	Y	Y
Hgb	Y	Y	Y	N	Y
Platelet	Y	Y	Y	Y	Y
Hct	Y	Y	Y	N	Y
iCa	Y	Y	Y	Y	Y
BGA	Y	Y	Y	Y	Y
Lactic acid	Y	Y	Y	N	Y
TEG	N	N	N	Y	N
TEG6S	Y	N	N	N	N
ROTEM	N	N	Y	N	N
Sampling frequency < 1 h	Y	N	Y	Y	Y

PT, prothrombin time; INR, international normalized ratio; APTT, activated partial thromboplastin time; Hgb, hemoglobin; Hct, hematocrit; iCa, ionized calcium; BGA, blood gas analysis; TEG, thromboelastography; TEG6S, Thromboelastograph 6s Hemostasis Analyzer System; ROTEM, rotational thromboelastometry.

**Table 5:** Use of adjunct therapies in massive transfusion protocols.

Adjunct Therapy	A	B	C	D	E
TXA	Y	Y	Y	Y	Y
Cryoprecipitate	Y	Y	Y	Y	Y
Fibrin concentrate	N	N	N	N	Y
PCC	N	N	N	N	Y

TXA, tranexamic acid; PCC, prothrombin complex concentrate.

**Table 6:** Termination criteria and transition strategy in massive transfusion protocols.

Item	A	B	C	D	E
Are termination criteria documented?	Y	Y	Y	Y	Y
Is the transition to goal-directed transfusion strategy specified?	Y	Y	Y	Y	Y

**Table 7:** Performance improvement monitoring elements in massive transfusion protocols.

Monitoring Element	A	B	C	D	E
Complications	Y	Y	Y	Y	Y
Time to first RBC transfusion	Y	N	N	N	N
Time to first plasma transfusion	Y	N	N	N	N
Time to first platelet transfusion	Y	N	N	N	N
Ratio of blood products transfused within 1 h of MTP activation	Y	N	N	N	N
Notification of relevant department within 1 h after MTP termination	Y	N	N	N	N
Rate of discarded blood products	Y	Y	Y	Y	Y
Documentation of regular in-department review	Y	N	N	N	Y
Documentation of regular multidisciplinary review	Y	N	N	N	N

RBC, red blood cell; MTP, massive transfusion protocol.

## Data Availability

Data presented in this study are available on request from the corresponding author due to ethical restrictions and patient privacy concerns.

## Supplementary Materials

The following supporting information can be downloaded at: https://www.mdpi.com/article/10.3390/healthcare13151848/s1, The COHTRA-K Questionnaire.

## Abbreviations

The following abbreviations are used in this manuscript:

ABC	Assessment of Blood Consumption
ACS	American College of Surgeons
COHTRA-K	Cohort for Optimal Hemostatic Transfusion in Trauma–Korea
ED	Emergency Department
FFP	Fresh frozen plasma
LTOWB	Low-titer O whole blood
MTP	Massive transfusion protocol
PI	Performance improvement
PRBC	Packed red blood cell
PROPPR	Pragmatic, Randomized Optimal Platelet and Plasma Ratios trial
RBC	Red blood cell
TQIP	Trauma Quality Improvement Program
TXA	Tranexamic acid
