# Supplementary material for: Toward Standardized Massive Transfusion Protocols: A Multicenter Evaluation of Practice Variability Within a National Trauma System
Source: Healthcare (Basel). 2025 Jul 29;13(15):1848. doi: 10.3390/healthcare13151848 (PMC12346504; doi:10.3390/healthcare13151848)
Supplement: Supplementary file 1 [file healthcare-13-01848-s001.zip › healthcare-3688173-supplementary.pdf]

Ajou University Hospital

Department of Trauma Surgery

The COHTRA-K Questionnaire

0. Please enter your affiliated institution:

____

1. Initiation Criteria

1-1. Is an objective activation criterion for the massive transfusion protocol (MTP) specified and documented?

○ Yes ○ No

1-2. If yes, please describe the activation criteria currently in use.

____

1-3. Is the physician's clinical judgment used as an activation criterion?

○ Yes ○ No

1-4. Is active bleeding requiring surgery or angioembolization included as an activation criterion?

○ Yes ○ No

1-5. Is the initiation of transfusion in the trauma bay used as an activation criterion?

○ Yes ○ No

1-6. Is there a rapid decision-making system in place for immediate activation?

○ Yes ○ No

1-7. Do you use any predictive score for MTP activation?

○ Yes ○ No

1-8. If yes, which score do you use?

[여기에 입력]

○ ABC score ○ TASH score ○ TBSS score ○ Shock index ○ Other : _________

1-9. Are blood products available within 15 minutes after MTP activation?

○ Yes ○ No

2. Fixed-Ratio Blood Component Batch

2-1. Please describe the basic fixed-ratio blood component used in your MTP.
Example: 4 RBC + 4 FFP + 1 Platelet pheresis

____

2-2. Are blood products stored in the trauma bay or emergency room (ER)?

○ Yes ○ No

2-3. Do you use type O red blood cells for initial transfusion? If yes, please specify the Rh type.

○ Yes, Rh+ type O RBCs are used.
○ Yes, Rh− type O RBCs are used.
○ No

2-4. Do you use type AB fresh frozen plasma for initial transfusion? If yes, please specify the Rh type.

○ Yes, Rh+ type AB FFPs are used.
○ Yes, Rh− type AB FFPs are used.
○ No

2-5. Do you use low-titer A FFP or thawed plasma?
○ Yes, we use low-titer A FFP.
○ Yes, we use thawed plasma.
○ No.

2-6. Are platelets included in the initial transfusion?

[여기에 입력]

○ Yes ○ No

2-7. Are platelets stored in the trauma bay?

○ Yes ○ No

2-8. Do you use a blood warmer during transfusion?

○ Yes ○ No

2-9. Do you use a rapid infusion device during transfusion?

○ Yes ○ No

3. Laboratory Monitoring

3-1. Are the types and frequency of laboratory tests specified in the MTP?

○ Yes ○ No

3-2. What is the sampling frequency?

____

3-3. Please check all laboratory monitoring parameters included in the MTP

○ PT (INR) ○ aPTT ○ Fibirnogen ○ Hb ○ Hct ○ Platelet ○ iCa ○ BGA

○ Lactic acid ○ Other : ________

3-4. Are the specific laboratory tests and their intervals clearly described in the MTP?

	Yes	No	Other
TEG			
TEG 6S			
ROTEM			

4. Adjunctive Therapies

4. Please indicate all adjuncts currently used or additionally incorporated in your massive

[여기에 입력]

transfusion protocol

○ TXA ○ Cryoprecipitate ○ Fibrin concentrate ○ PCC ○ Other : _________

5. Termination Criteria

5-1. Are objective criteria for termination of the MTP specified and documented?

○ Yes ○ No

5-2. Is the transition to a goal-directed transfusion strategy specified in the protocol?

○ Yes ○ No

6. Performance Improvement Monitoring

6. Do you perform Performance Improvement monitoring on the following items during MTP implementation?

	Yes No Other
Complications	
Time to first RBC transfusion	
Time to first plasma transfusion	
Time to first platelet transfusion	
Ratio of blood products transfused within 1 h of MTP activation	
Notification of relevant department within 1 h after MTP termination	
Rate of discarded blood products	
Documentation of regular in-department review	
Documentation of regular multidisciplinary review	

[여기에 입력]
